# The role of YTH domain containing 2 in epigenetic modification and immune infiltration of pan‐cancer

**DOI:** 10.1111/jcmm.16818

**Published:** 2021-07-27

**Authors:** Chiyuan Zhang, Cuishan Guo, Yan Li, Ling Ouyang, Qi Zhao, Kuiran Liu

**Affiliations:** ^1^ Department of Obstetrics and Gynecology Shengjing Hospital of China Medical University Shenyang China; ^2^ School of Computer Science and Software Engineering University of Science and Technology Liaoning Anshan China

**Keywords:** diagnosis, immune infiltration, m^6^A, prognosis, YTHDC2

## Abstract

YTH domain containing 2 (YTHDC2) is the largest N6‐Methyladenosine (m^6^A) binding protein of the YTH protein family and the only member containing ATP‐dependent RNA helicase activity. For further analysing its biological role in epigenetic modification, we comprehensively explored YTHDC2 from gene expression, genetic alteration, protein‐protein interaction (PPI) network, immune infiltration, diagnostic value and prognostic value in pan‐cancer, using a series of databases and bioinformatic tools. We found that YTHDC2 with Missense mutation could cause a different prognosis in uterine corpus endometrial carcinoma (UCEC), and its different methylation level could lead to a totally various prognosis in adrenocortical carcinoma (ACC), cervical squamous cell carcinoma and endocervical adenocarcinoma (CESC), lung squamous cell carcinoma (LUSC) and UCEC. The main molecular mechanisms of YTHDC2 focused on catalytic activity, helicase activity, snRNA binding, spliceosome and mRNA surveillance. Additionally, YTHDC2 was notably correlated with tumour immune infiltration. Moreover, YTHDC2 had a high diagnostic value for seven cancer types and a prognostic value for brain lower grade glioma (LGG), rectum adenocarcinoma (READ) and skin cutaneous melanoma (SKCM). Collectively, YTHDC2 plays a significant role in epigenetic modification and immune infiltration and maybe a potential biomarker for diagnosis and prognosis in certain cancers.

## 1 INTRODUCTION

In recent years, as the most abundant mRNA modification in eukaryotic cells,^1^ m^6^A RNA modification is widely recognized as an essential epigenetic modification in many biological processes by the dynamic adjustment of gene expression and receives considerable attention. It is well established that m^6^A modification is crucial for various pathological processes, including cancer initiation and progression.^2^, ^3^, ^4^, ^5^, ^6^ Although the dedicated methyltransferases (writers) and demethylases (erasers) regulate the reversibility of m^6^A modification, which does not alter the matching and coding of bases, m^6^A binding proteins (readers) are vital to the fate of mRNAs through specific recognizing m^6^A and binding function during various bioprocesses, such as RNA splicing, export, translation and decay,^1^ widely affecting gene expression at different levels. Among diverse m^6^A binding proteins, the YTH protein family has been identified to play a critical role in m^6^A modification, including YTHDF1, YTHDF2, YTHDF3, YTHDC1 and YTHDC2. YTHDF1 contributes to enhance the initiation of translating and promote protein synthesis through interacting with eIF3.^7^ YTHDF2 can mediate m^6^A‐dependent mRNA decay.^8^ YTHDF3 takes an important part in the translation process of m6A‐containing mRNAs through the sequential recruitment of other effectors.^9^ YTHDC1 regulates gene splicing and modulate the exportation of m6A‐modified mRNA.^2^ YTHDC2 serves as the largest protein member (approximately 160 kD) in the YTH protein family. YTHDC2 is distinct from other YTH proteins because of its special helicase domains. YTHDC2 prefers to bind to the conserved m^6^A‐modified motifs and executives the function of m^6^A reader by enhancing the translation efficiency and decreasing the mRNA abundance.^10^, ^11^, ^12^, ^13^ Previous studies have shown that YTHDC2 has a crucial effect on cancer metastasis through increased translation efficiency of HIF‐1α in colon cancer patients^14^ and contributes significantly in the proliferation of hepatocellular carcinoma cells.^15^ YTHDC2 expression has been identified to be associated with prognosis, apoptosis activation and ubiquitin‐mediated proteolysis in Head and Neck squamous cell carcinoma (HNSCC).^16^

However, although certain studies have been carried out on YTHDC2, no single study exists which could overall evaluate its effects on considerable types of cancers. In light of increasing concern of pan‐cancer genetic analysis, which will be beneficial to assess diagnostic and clinical prognostic values of genes on the whole level, we explored YTHDC2 expression in pan‐cancer to further identify the influence of YTHDC2 on tumour promotion and suppression. As a result, we observed that YTHDC2 expressed significantly differently in cancers compared with normal tissues and was down‐regulated in most tumours. We also found that YTHDC2 not only showed a high diagnostic value in predicting cancers containing cholangiocarcinoma (CHOL), LUSC, thyroid carcinoma (THCA), ovarian serous cystadenocarcinoma (OV), SKCM, testicular germ cell tumours (TGCT) and uterine carcinosarcoma (UCS), but also displayed its prognostic value in LGG, READ and SKCM. Furthermore, YTHDC2 with genetic alteration was significantly correlated with the prognosis of UCEC, ACC, LUSC and CESC. In addition, YTHDC2 was identified to play an important role in tumour cell immune infiltration. The pan‐cancer analysis may contribute to uncovering the distinct roles of YTHDC2 in the varying cancer promotion or suppression and provide evidence for more directional clinical and experimental research in individual cancers.

## 2 MATERIALS AND METHODS

### 2.1 Gene expression analysis

The RNA‐seq data and relevant clinical data in level 3 transcripts per million reads (TPM) of 15,776 samples across 33 tumour types from The Cancer Genome Atlas (TCGA; https://portal.gdc.cancer.gov/; tcga_RSEM_gene_tpm) and the Genotype‐Tissue Expression (GTEX) database (dataset ID: gtex_RSEM_gene_tpm) were downloaded by UCSC XENA (https://xenabrowser.net/datapages/). Then, the RNA‐seq data in TPM format were converted into log_2_ format for expression comparison between samples. R software v3.6.3 was used for statistical analysis, and ggplot2 package was for visualization. The Wilcoxon rank sum test detected two sets of data, and *p* < 0.05 was considered statistically significant. (ns, *p* ≥ 0.05; *, *p* < 0.05; **, *p* < 0.01; ***, *p* < 0.001).^17^ We also displayed the differential expression of YTHDC2 between tumours and adjacent normal tissues using Tumor Immune Estimation Resource (TIMER).

### 2.2 Genetic alteration analysis

Genomic profiles, including the alteration frequency, mutation type and Copy number alterations (CNA) across all TCGA tumours, were calculated by using the cBioPortal web (https://www.cbioportal.org/). Kaplan‐Meier plots with log‐rank *p*‐value was generated by obtaining the data on the overall survival (OS), progression‐free survival (PFS), disease‐free survival (DFS) and disease‐specific survival (DSS) of UCEC cases with or without YTHDC2 genetic alteration.^18^, ^19^ Additionally, we used Gene Set Cancer Analysis (GSCA; http://bioinfo.life.hust.edu.cn/),^20^ which is an integrated genomic and immunogenomic web‐based platform for gene set cancer research to assess the correlation between YTHDC2 methylation and prognosis in cancers.

### 2.3 PPI network analysis

The online STRING (https://string‐db.org/) tool provides investigators with systematic and comprehensive functional annotation tools for identifying the biological significance of an extensive list of genes. We obtained 50 YTHDC2‐binding proteins by setting the following main parameters: minimum required interaction score (‘medium confidence [0.400]’), meaning of network edges (‘evidence’), max number of interactors to show (‘no more than 50 interactors’ in 1st shell) and active interaction sources (‘Experiments, Text mining, Databases’). Then, Cytoscape (version 3.7.2) was applied for visualization of PPI networks.

### 2.4 Functional and pathway enrichment analysis

In our study, Gene ontology (GO) and Kyoto Encyclopedia of Genes and Genomes (KEGG) enrichment analyses were conducted for YTHDC2‐binding proteins using ggplot2 package for visualization and cluster Profiler package for statistical analysis.^21^, ^22^

### 2.5 Distribution of YTHDC2 expression in molecular subtypes and immune subtypes of cancers

TISIDB (cis.hku.hk/TISIDB/)^23^ is a web portal for tumour and immune system interaction, which integrates multiple heterogeneous data types. We explored the association between YTHDC2 expression and molecular subtypes or immune subtypes across TCGA tumours from TISIDB database.

### 2.6 Immune infiltration analysis

Tumor immune estimation resource^24^, ^25^, ^26^ is a comprehensive resource for systematical analysis of immune infiltrates across diverse cancer types. The abundances of six immune infiltrates (B cells, CD4+ T cells, CD8+ T cells, neutrophils, macrophages and dendritic cells) are estimated by TIMER algorithm. We explored the association between YTHDC2 expression and immune infiltration across TCGA tumours. Additionally, we used GSCA^20^ to assess the correlation between YTHDC2 expression and immune infiltration. Also, we used immunedeconv package for reliable immune score evaluation. Furthermore, we examined the correlation between YTHDC2 expression and immune checkpoint‐related genes (SIGLEC15,^27^ IDO1,^28^ CD274,^29^ HAVCR2,^30^ PDCD1,^31^ CTLA4,^26^ LAG3^32^ and PDCD1LG2^33^) using R software. The horizontal axis represents the expression of immune checkpoint‐related genes, and the vertical axis represents different tumour tissues. Different colours represent correlation coefficients, blue colour represents positive correlation, while red colour represents negative correlation, and the darker colour represents the stronger correlation (*p <  0.05, **p < 0.01, ***p < 0.001).

### 2.7 Diagnostic value analysis

The clinicopathological parameters of 33 tumour patients from TCGA database and the corresponding normal tissue data from GTEX database were extracted to assess the diagnostic value of YTHDC2 by receiver operating characteristic (ROC) curve using pROC package for analysis and ggplot2 package for visualization. Note: The area value under the ROC curve is between 0.5 and 1. The closer the area under the curve (AUC) is 1, the better the diagnostic effect is. AUC in 0.5–0.7 has a low accuracy, AUC in 0.7–0.9 has a certain accuracy, and AUC above 0.9 has a high accuracy.

### 2.8 Survival prognosis analysis

Kaplan‐Meier plots were presented to assess the relationship between YTHDC2 expression level and prognosis for various cancer types, survminer package was used for visualization, and survival package was used for statistical analysis of survival data. The Log‐rank test was used in the hypothesis test, and *p* < 0.05 is considered statistically significant.

## 3 RESULTS

### 3.1 Analysis of YTHDC2 expression in cancers

**FIGURE 1:**
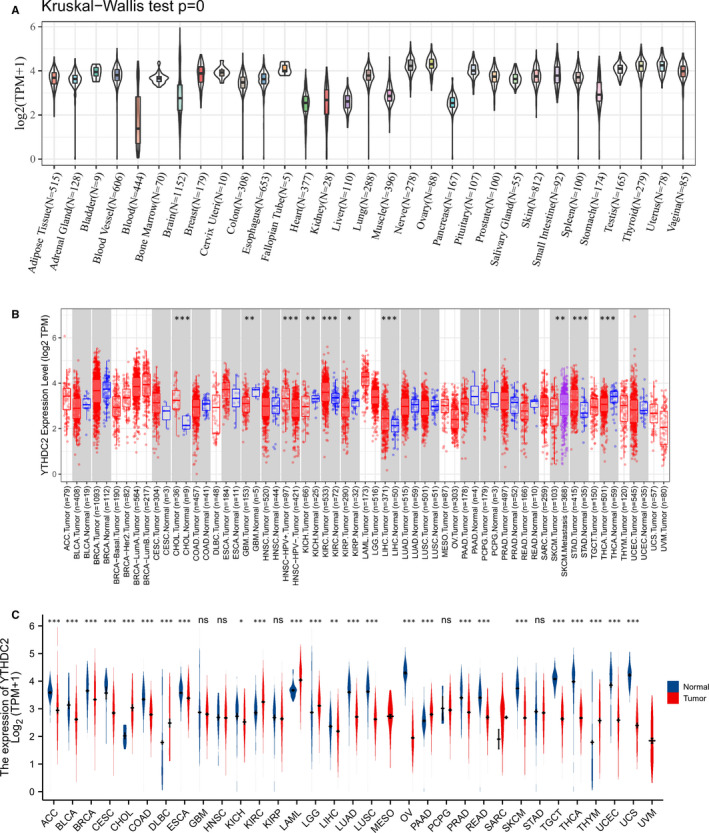
Expression level of YTHDC2 gene in tumours and normal tissues. (A) YTHDC2 expression in normal tissues. (B) YTHDC2 expression in TCGA tumours and adjacent normal tissues. (C) YTHDC2 expression in TCGA tumours and normal tissues with the data of the GTEX database as controls. *p < 0.05, **p < 0.01, ***p < 0.001, the asterisk represents the degree of importance (*p). The significance of the two groups of samples passed the Wilcox test

We displayed YTHDC2 expression in normal tissues from GTEX database (Figure 1A), the different expression levels in TCGA tumours and adjacent normal tissues from TIMER (Figure 1B), and the distribution of YTHDC2 expression in tumours with the data of the GTEX database as controls (Figure 1C). The expression level of YTHDC2 was significantly upregulated in CHOL, lymphoid neoplasm diffuse large B‐cell lymphoma (DLBC), kidney renal clear cell carcinoma (KIRC), acute myeloid leukaemia (LAML), LGG, pancreatic adenocarcinoma (PAAD) and thymoma (THYM). On the contrary, the expression level of YTHDC2 was notably down‐regulated in ACC, bladder urothelial carcinoma (BLCA), breast invasive carcinoma (BRCA), CESC, colon adenocarcinoma (COAD), oesophageal carcinoma (ESCA), kidney chromophobe (KICH), liver hepatocellular carcinoma (LIHC), lung adenocarcinoma (LUAD), LUSC, OV, prostate adenocarcinoma (PRAD), rectum adenocarcinoma (READ), SKCM, TGCT, THCA, UCEC, and UCS.

### 3.2 Genetic alteration analysis of YTHDC2

**FIGURE 2:**
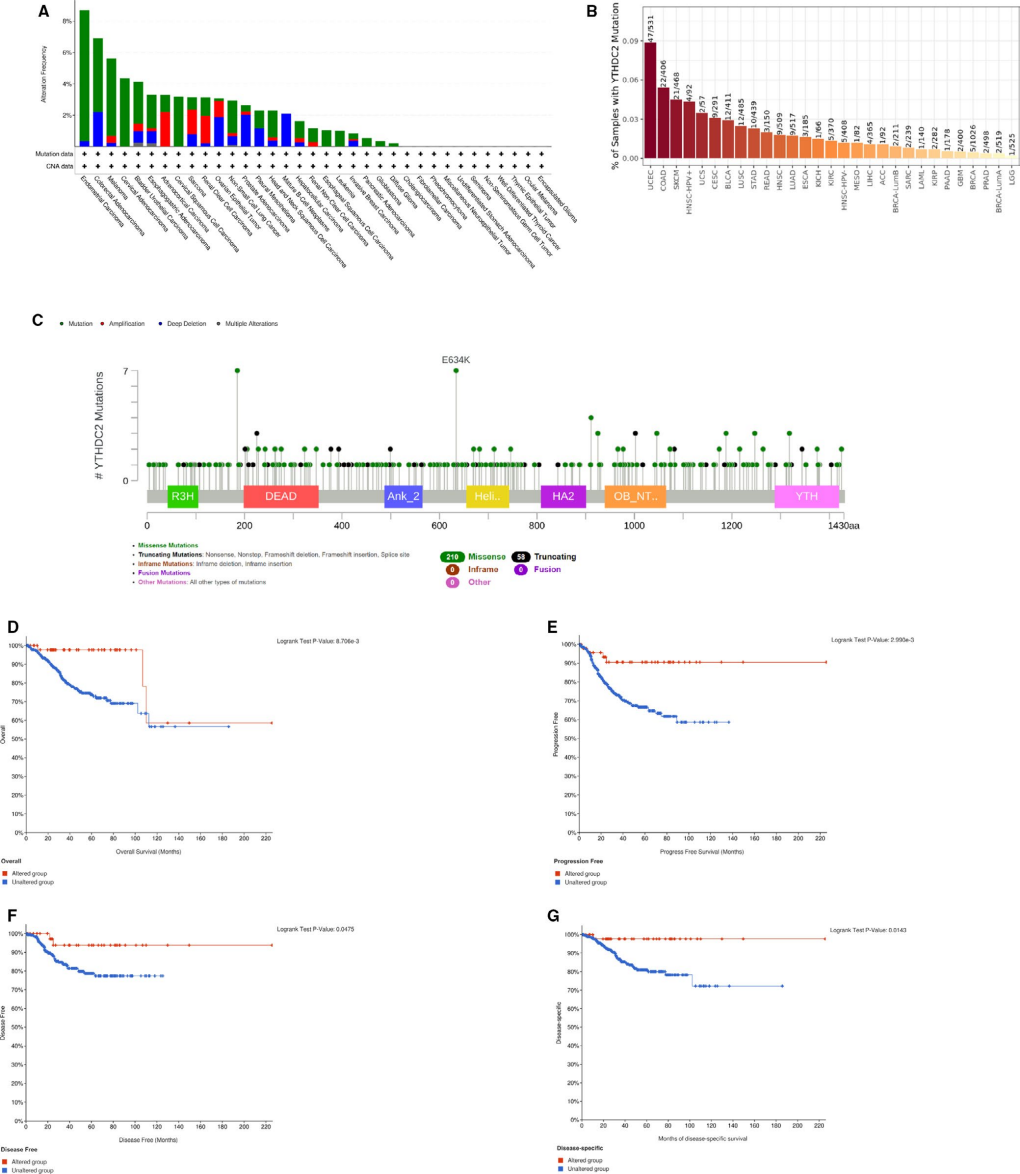
The genetic alteration status of YTHDC2 in pan‐cancer. (A) The alteration frequency of YTHDC2 containing mutation, amplification, deep deletion and multiple alterations. (B) Number of samples with YTHDC2 mutation. (C) The mutation site of YTHDC2. (D‐G) The OS, PFS, DFS and DSS of UCEC between altered group and unaltered group of YTHDC2

**FIGURE 3:**
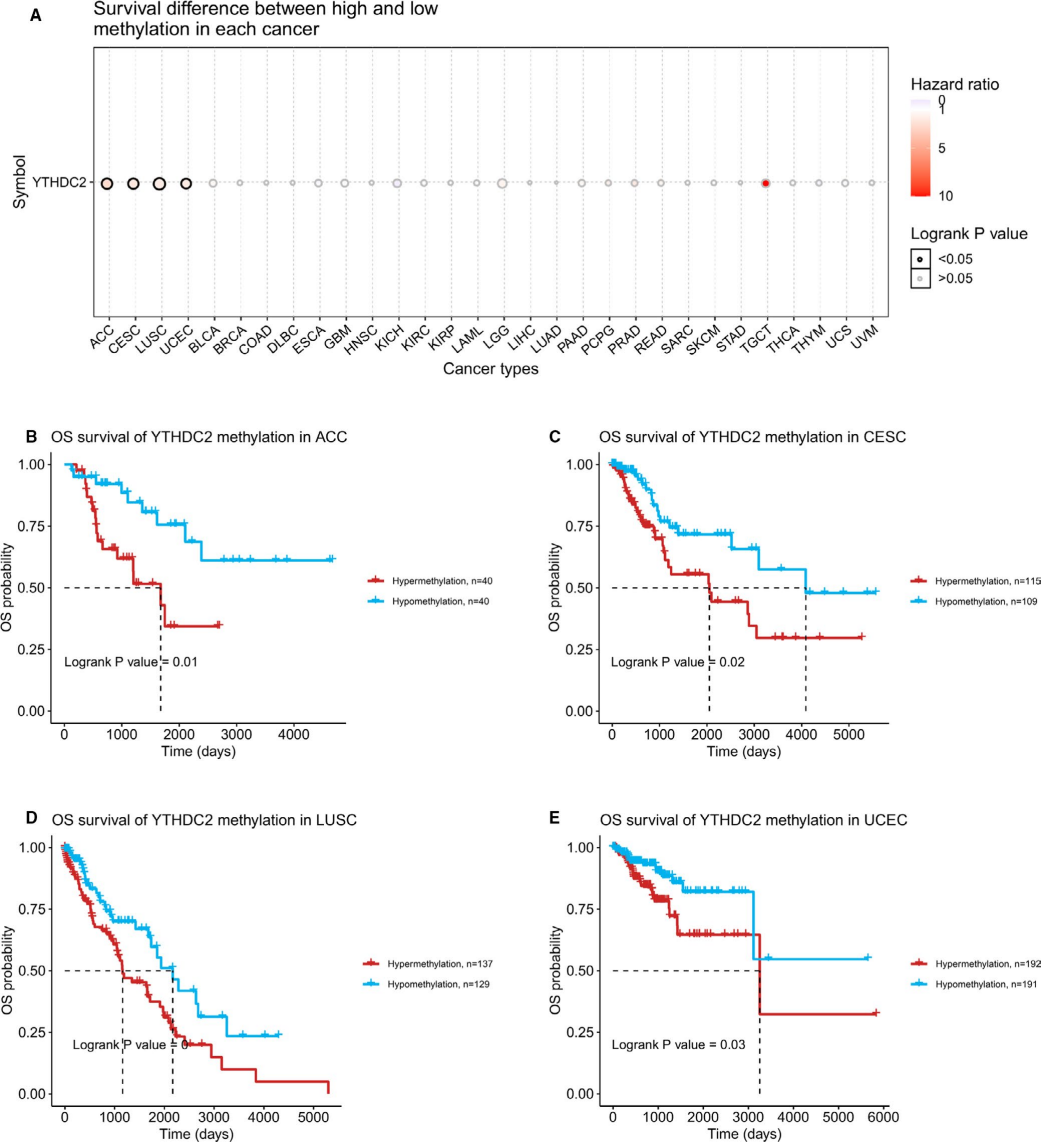
Correlations between YTHDC2 methylation and OS. (A) Survival difference between high and low methylation of YTHDC2 in pan‐cancer. (B) in ACC. (C) in CESC. (D) in LUSC. (E) in UCEC

The results of the preliminary analysis of the genetic alteration status of YTHDC2 are presented in Figure 2. The results indicated that mutation as the highest alteration frequency (8.36%) occurred in patients with UCEC. Furthermore, samples of CESC with genetic alteration only had mutation of YTHDC2, with the alteration frequency showed 4.35% and 3.19% respectively. Besides, patients with ACC had the higher alteration frequency (2.2%) of amplification than all the other tumours, and all mature B‐cell neoplasms cases with genetic alteration had deep deletion (2.08%) of YTHDC2 (Figure 2B). We also observed 268 mutations of YTHDC2 presented in Figure 2C, including types, sites and case numbers of mutations and found that the key gene alteration type was the missense mutation. In Figure 2D‐G, we explored that the prognosis of UCEC patients with YTHDC2 genetic alteration was better than the cases without YTHDC2 genetic alteration in PFS, DFS and DSS. However, patients with YTHDC2 hypermethylation had a worse OS than patients with YTHDC2 hypomethylation in ACC, CESC, LUSC and UCEC (Figure 3).

### 3.3 Enrichment analysis of YTHDC2‐related genes in cancers

**FIGURE 4:**
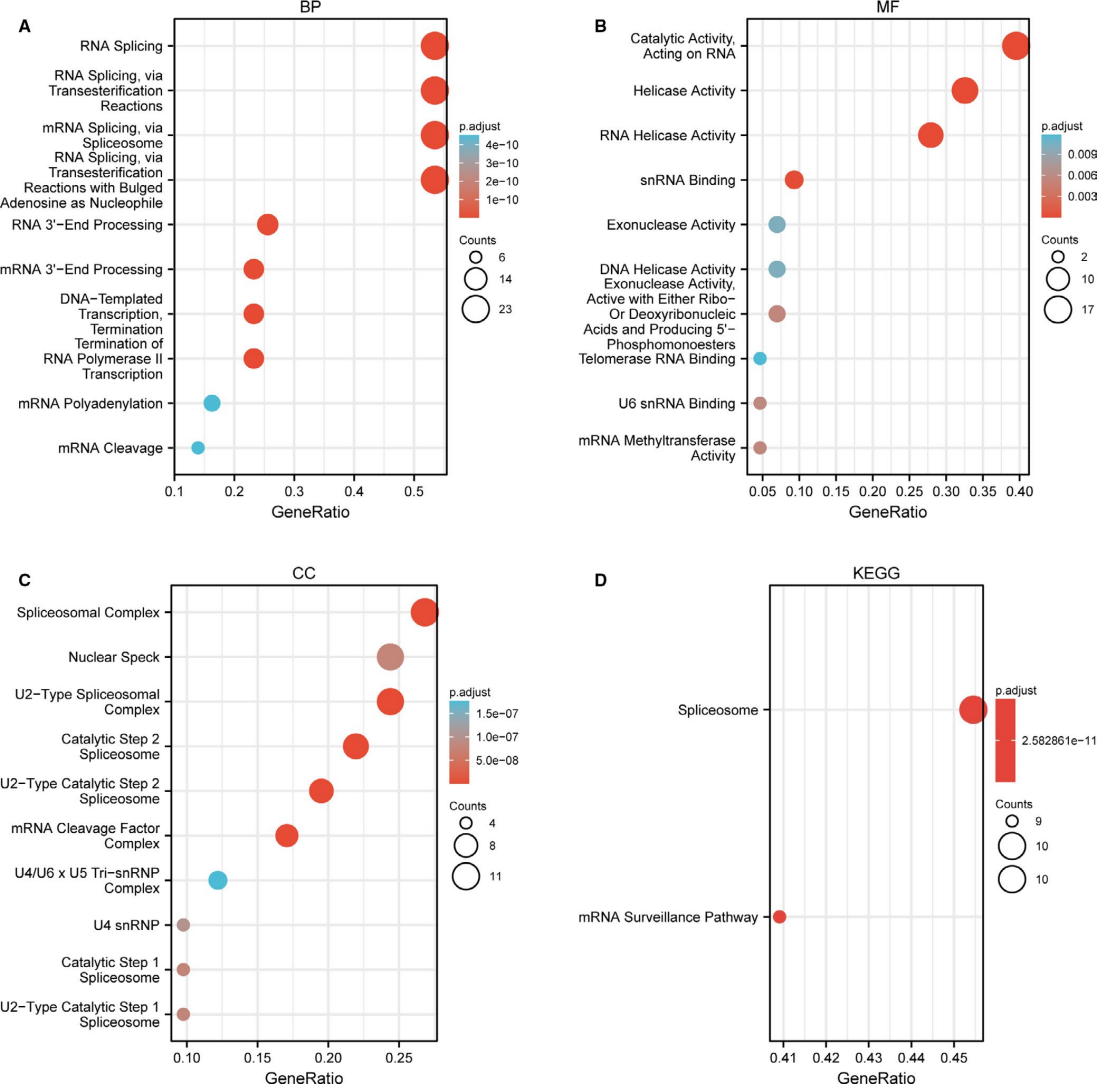
Gene ontology and KEGG analysis of 50 targeted binding proteins of YTHDC2. (A) BP enrichment analysis. (B) MF enrichment analysis. (C) CC enrichment analysis. (D) The KEGG pathway analysis

We screened out 50 targeted binding proteins of YTHDC2 using STRING's website and showed the interaction network by cytoscape (Figure S1). Then, we conducted the GO and KEGG pathway enrichment analyses to further investigate the molecular mechanism of YTHDC2 (Figure 4, Figure S2). The results suggested that the biological process (BP) was primarily involved in the aspect of RNA Splicing, RNA3'‐end processing, DNA‐templated transcription termination and termination of RNA polymerase II transcription; the major aspects of molecular function (MF) contained catalytic activity acting on RNA, helicase activity, and snRNA binding; the cellular component (CC) mainly included Spliceosomal Complex, Nuclear Speck, U2‐Type Spliceosomal Complex, Catalytic Step 2 Spliceosome, U2−Type Catalytic Step 2 Spliceosome, and mRNA Cleavage Factor Complex. Additionally, KEGG enrichment analysis was mainly linked to the spliceosome and mRNA surveillance pathway.

### 3.4 Molecular subtypes and immune subtype's analysis in cancers

We used TISIDB database to show the correlation between YTHDC2 expression and molecular subtypes or immune subtypes across TCGA tumours. As shown in Figure S3, YTHDC2 expression was significantly correlated with molecular subtypes in ACC, BRCA, COAD, HNSC, LGG, pheochromocytoma and paraganglioma (PCPG), PRAD, stomach adenocarcinoma (STAD) and UCEC.

A total of six immune subtype were compared with YTHDC2 expression across diverse cancers, including C1 (wound healing), C2 (IFN‐gamma dominant), C3 (inflammatory), C4 (lymphocyte depleted), C5 (immunologically quiet) and C6 (TGF‐b dominant), and YTHDC2 was notably associated with immune subtypes of nine cancer types, including BLCA, BRCA, KIRC, LIHC, LUAD, PAAD, SKCM, STAD and UCEC (Figure S4).

### 3.5 Immune infiltration analysis in cancers

We used the TIMER to assess the correlation between YTHDC2 expression and immune infiltration in cancers. The results showed that YTHDC2 expression was closely related to tumour purity in 17 types of cancers. Among them, YTHDC2 had negative associations with tumour purity in seven cancer types, including BRCA‐Basal, READ, COAD, KIRC, HNSC‐HPVneg, LUAD, together with OV. YTHDC2 expression was significantly positively correlated with the infiltration levels of B Cell, CD8+ T cell, CD4+ T cell, macrophage, Neutrophil, and Dendritic Cell in BRCA‐Basal, COAD, KIRC, and LUAD; with the infiltration levels of CD8+ T cell, CD4+ T cell, macrophage, neutrophil and dendritic cell in HNSC‐HPVneg; with the infiltration levels of B cell, CD8+ T cell, neutrophil and dendritic cell in READ; with the infiltration levels of CD8+ T cell, neutrophil and dendritic cell in OV (Figure S5). Yet, YTHDC2 was positively associated with tumour purity in 10 cancer types, such as BRCA, SKCM, SKCM‐primary, glioblastoma multiforme (GBM), BRCA‐Luminal, BLCA, Sarcoma (SARC), TGCT, LGG and ACC (Figure S6). Meanwhile, we performed a spearman correlation analysis heat map of immune score and YTHDC2 gene expression in multiple tumour tissues (Figure S7). The results showed that YTHDC2 expression was significantly positively correlated with the infiltration levels of CD8+ T cell, CD4+ T cell, neutrophil, myeloid dendritic cell, macrophage and B cell in five types of cancer, containing COAD, KIRC, LIHC, LUAD and PAAD. Then, we examined the associations between YTHDC2 expression and immune infiltrates in GSCA (Figure S8). In COAD, YTHDC2 showed a positive correlation with the infiltration levels of mucosal associated invariant T cell (MAIT), T follicular helper cell (Tfh) and induced regulatory T cell (iTreg), but a negative correlation with the infiltration levels of CD8 naïve T cell (CD8_native). In KIRC, YTHDC2 showed a positive correlation with the infiltration levels of CD4 T cell (CD4_T), CD8 T cell (CD8_T), central memory T cell (Central_memory), cytotoxic T cell (Cytotoxic), dendritic cells (DC), exhausted T cell (Exhausted), Infiltration Score, macrophage, monocyte, Tfh, T helper type 1 (Th1), type 1 regulatory T cell (Tr1), iTreg and natural Treg (nTreg), but a negative correlation with the infiltration levels of CD4 naïve T cell (CD4_native), CD8_native, neutrophil, T helper type 17 (Th17) and T helper type 2 (Th2). In LIHC, YTHDC2 was positively correlated with the infiltration levels of CD4_T, CD4_native, central memory T cell (Central_memory) and iTreg, but negatively correlated with the infiltration levels of B cell, CD8_T, cytotoxic, effector memory T cell (Effector_memory), Gamma delta T cell (Gamma_delta), Infiltration Score and Natural killer T cell (NKT). In LUAD, YTHDC2 was positively correlated with the infiltration levels of CD4_T, Central_memory, Infiltration Score, Tfh, Tr1 and iTreg, but negatively correlated with the infiltration levels of Effector_memory, exhausted, neutrophil and nTreg. In PAAD, YTHDC2 was positively correlated with the infiltration levels of CD4_T, Central_memory, cytotoxic, Gamma_delta, MAIT, NK, Tfh, Tr1 and iTreg, while negatively correlated with the infiltration levels of macrophage, monocyte, neutrophil, Th17 and Th2.

**FIGURE 5:**
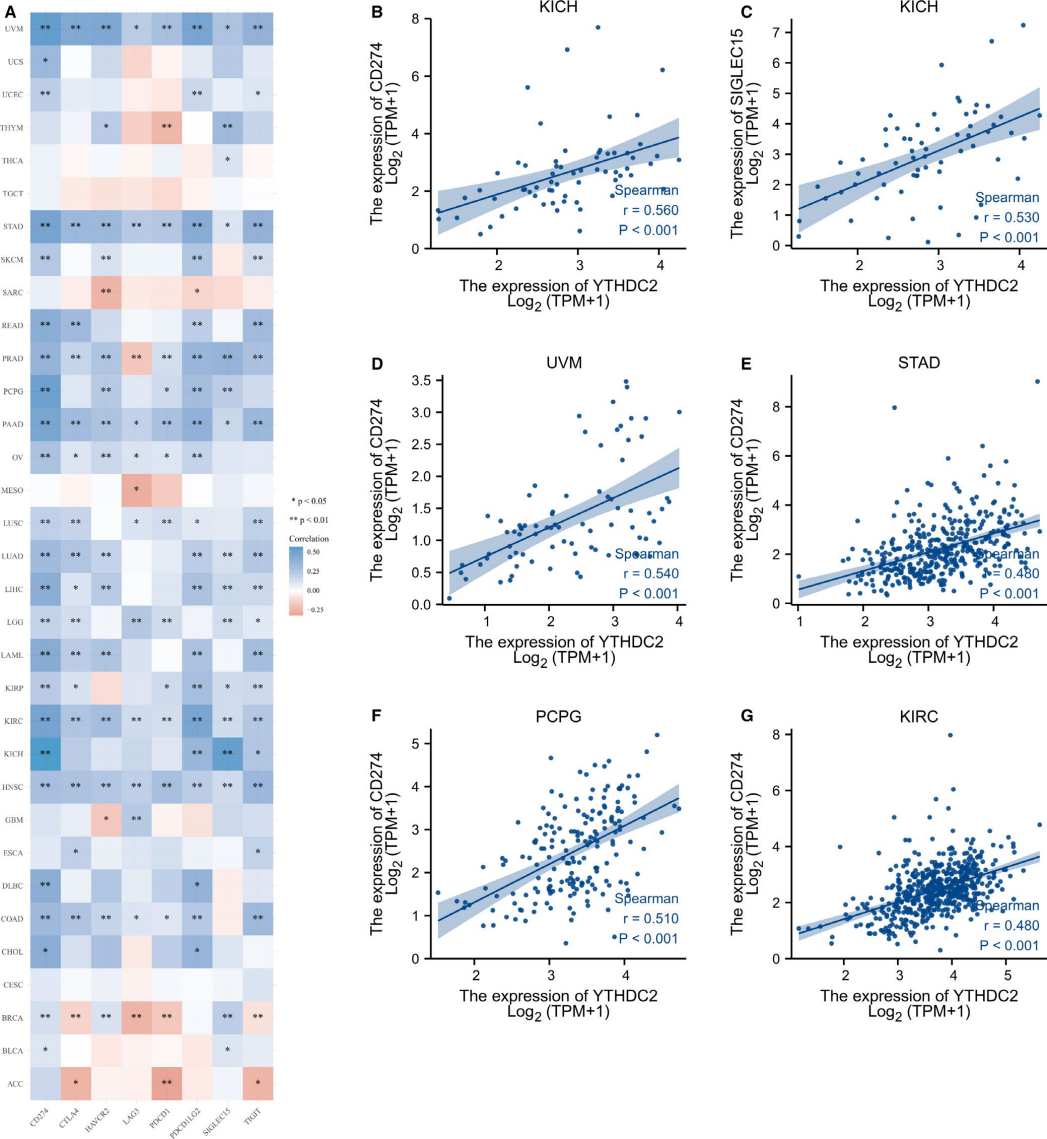
Correlations between YTHDC2 expression and immune checkpoint‐related genes. (A) The expression heat map of immune checkpoint‐related genes in different tumour tissues and the correlations with YTHDC2. (B) Correlation between YTHDC2 and CD274 in KICH. (C) Correlation between YTHDC2 and SIGLECT15 in KICH. (D) Correlation between YTHDC2 and CD274 in UVM. (E) Correlation between YTHDC2 and CD274 in STAD. (F) Correlation between YTHDC2 and CD274 in PCPG. (G) Correlation between YTHDC2 and CD274 in KIRC

We further explored the correlation between YTHDC2 and eight immune checkpoint‐related genes and found that YTHDC2 was significantly associated with the expression of SIGLEC15, IDO1, CD274, HAVCR2, PDCD1, CTLA4, LAG3 or PDCD1LG2 in almost all types of cancers except for CESC and TGCT (Figure 5).

### 3.6 Diagnostic value of YTHDC2 in cancers

**FIGURE 6:**
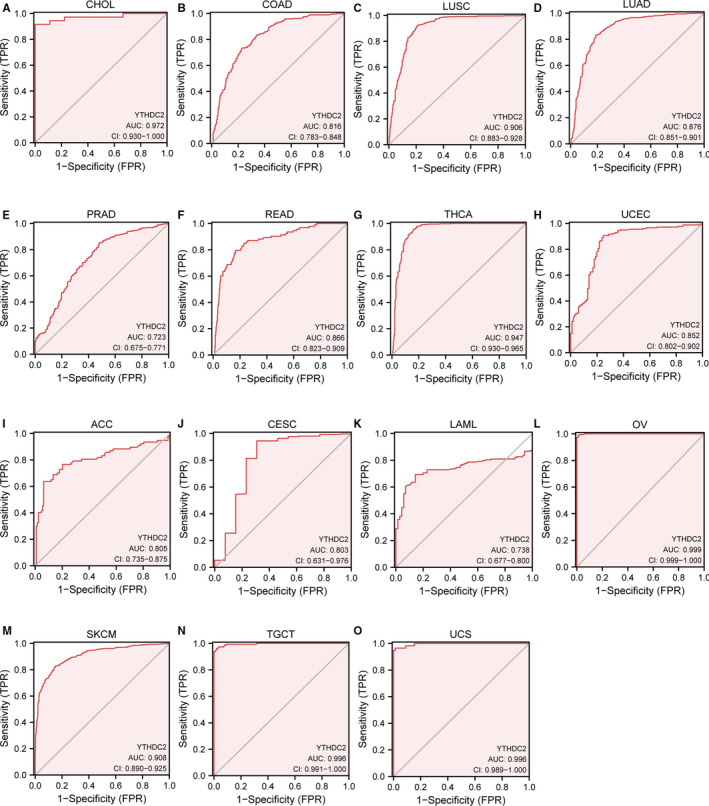
Receiver operating characteristic curve for YTHDC2 expression in pan‐cancer. (A) CHOL. (B) COAD. (C) LUSC. (D) LUAD. (E) PRAD. (F) READ. (G) THCA. (H) UCEC. (I) ACC. (J) CESC. (K) LAML. (L) OV. (M) SKCM. (N) TGCT. (O) UCS

We performed the ROC curve analysis to evaluate the diagnostic value of YTHDC2. As shown in Figure 6, YTHDC2 had a certain accuracy in predicting normal and tumour outcomes in 15 types of tumours, including CHOL, COAD, LUSC, LUAD, PRAD, READ, THCA, UCEC, ACC, CESC, LAML, OV, SKCM, TGCT and UCS. It is worth noting that YTHDC2 had a high diagnostic value for seven types of tumours, including CHOL (AUC = 0.972), LUSC (AUC = 0.906), THCA (AUC = 0.947), OV (AUC = 0.999), SKCM (AUC = 0.908), TGCT (AUC = 0.996) and UCS (AUC = 0.996).

### 3.7 Survival prognosis of YTHDC2 in cancers

**FIGURE 7:**
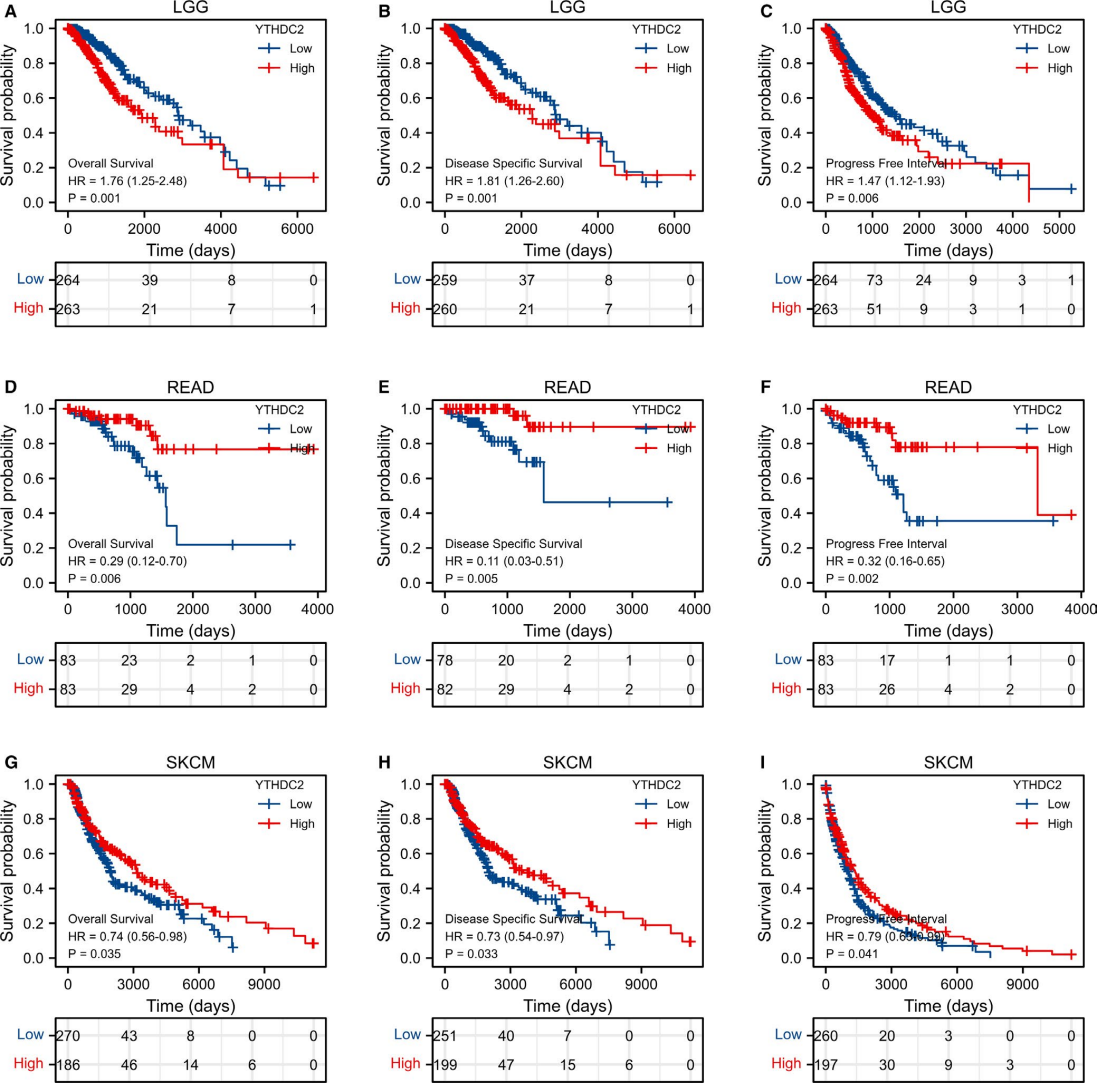
The OS, DSS and PFI of three types of cancers is significantly correlated with YTHDC2 expression. (A‐C) LGG. (D‐F) READ. (G‐I) SKCM

YTHDC2 expression was significantly correlated with the prognosis of LGG, READ and SKCM (Figure 7). For LGG, Cox regression results showed that the difference in survival time distribution in group was statistically significant for OS (Hazard Ratio [HR] = 1.76, 95% Confidence Interval (CI): 1.25–2.48, *p* = 0.001], DSS (HR = 1.81, 95% CI: 1.26–2.60, *p* = 0.001) and progress‐free interval (PFI; HR = 1.47, 95% CI: 1.12–1.93, *p* = 0.006), illustrating that the high expression level of YTHDC2 had a worse prognosis. In contrast, for READ, Cox regression results showed that the difference in survival time distribution in group was statistically significant for OS [HR = 0.29, 95% CI: 0.12–0.70, *p* = 0.006], DSS (HR = 0.11, 95% CI: 0.03–0.51, *p* = 0.005) and PFI (HR = 0.32, 95% CI: 0.16–0.65, *p* = 0.002), illustrating that the high expression level of YTHDC2 had a better prognosis. For SKCM, Cox regression results showed that the difference in survival time distribution in group was significant for OS (HR = 0.74, 95% CI: 0.56–0.98, *p* = 0.035), DSS (HR = 0.73, 95% CI: 0.54–0.97, *p* = 0.033) and PFI (HR = 0.79, 95% CI: 0.63–0.99, *p* = 0.041), indicating that the high expression level of YTHDC2 had a better prognosis.

## 4 DISCUSSION

YTHDC2 serves as not only the multi‐domain m^6^A reader containing the YTH domain, the R3H domain, the Helicase domain and the DUF1065 domain, but also the largest YTH domain‐containing protein owning 1430 amino acid sequences, especially possesses ATP‐dependent RNA helicase activity.^12^, ^13^ The YTH domain mediates RNA‐binding, recognizes and binds m^6^A‐containing RNAs. Recent studies have reported that YTHDC2 plays a key role in meiosis and spermatogenesis.^10^, ^11^, ^12^, ^13^ Existing research has recognized the critical part played by YTHDC2 in the initiation and progression of diseases including cancers. For instance, YTHDC2 could suppress mRNA stability of lipogenic genes by finding m^6^A methylated sites and binding their mRNAs in order to regulate lipogenic genes expression in the liver, as a result of affecting the metabolism of liver lipids and suppressing liver steatosis^34^; YTHDC2 might be a potential molecular target in terms of the susceptibility of pancreatic cancer as well as a valuable marker for early detection^35^; YTHDC2 was identified to be a potential therapeutic target in radio sensitization of nasopharyngeal carcinoma (NPC) by favouring the translation efficiency of IGF1R mRNA to promote radiotherapy resistance^36^; the loss of YTHDC2 contributed to the proliferation in oesophageal squamous‐cell carcinoma.^37^ However, in reviewing the literature, no study was found to comprehensively assess its distinct influence on pan‐cancer.

In the present study, firstly, we examined YTHDC2 expression in TCGA tumours and found that YTHDC2 was significantly down‐regulated in the majority of cancers than in the normal samples, including ACC, BLCA, BRCA, CESC, COAD, ESCA, KICH, LIHC, LUAD, LUSC, OV, PRAD, READ, SKCM, TGCT, THCA, UCEC and UCS, but was up‐regulated in CHOL, DLBC, KIRC, LGG, PAAD, LAML and THYM. The results show that there is a considerable difference in YTHDC2 expression in the vast majority of malignant tumours, and moreover, YTHDC2 is tumour‐suppressive in most cancers and is oncogenic in a few tumours, respectively. Like the dual role of m^6^A methylation playing in human cancers, on the one hand, it could accelerate the progression of certain tumours through positively affecting m^6^A modification; on the other hand, it may have a protective effect on tumour suppression. It is of great importance that YTHDC2 expression differs in different molecular subtypes of cancers, including ACC, BRCA, COAD, HNSC, LGG, PCPG, PRAD, STAD and UCEC, suggesting that it could be more meaningful for YTHDC2 study focusing on a certain molecular subtype of cancers.

Then, we observed that the missense mutation of YTHDC2 was the primary type, and the highest incidence of that occurred in UCEC patients. Interestingly, we also found that there was a meaningful difference between the altered group of YTHDC2 and the unaltered group in PFS, DFS and DSS, indicating that the missense mutation of YTHDC2 might be essential for the prognosis of UCEC. Meanwhile, we confirmed that the YTHDC2 methylation level was closely related to the prognosis of patients with ACC, CESC, LUSC and UCEC, identifying the potential prognostic value of YTHDC2 methylation in the above cancers.

As previous studies demonstrated, YTHDC2 can promote the efficiency of translation of mRNA secondary structures probably due to RNA helicase activity,^38^ and also, RNA‐binding domains contribute to the interrelationship between m^6^A‐containing mRNAs and the ribosomes. Additionally, YTHDC2 favours the RNA degradation for the purpose of managing the stability of mRNAs.^39^ Thus, we conducted GO and KEGG analysis of 50 targeted binding proteins of YTHDC2 to further investigate its molecular mechanism, revealing that the catalytic activity, helicase activity, snRNA binding, spliceosome and mRNA surveillance were mainly involved in BP, MF and enrichment pathway.

Prior studies have noted the crucial correlation between m^6^A modification and tumour microenvironment, implying that diverse m^6^A modification patterns play a vital role in the multiplicity and perplexity of tumour microenvironment.^40^, ^41^ According to Spearman correlation analysis of immune score and YTHDC2 gene expression, our study found that YTHDC2 was critically involved in the immune infiltration of cancers, especially in COAD, KIRC, LIHC, LUAD and PAAD, due to the positive correlation between YTHDC2 expression and the infiltration levels of CD8+ T cell, CD4+ T cell, neutrophil, myeloid dendritic cell, macrophage and B cell. In addition, the results showed that YTHDC2 expression was significantly positively correlated with B cell in 21 types of cancers, with CD4+ T cell in 17 types of cancers, with CD8+ T cell in 27 types of cancers, with dendritic cell in 23 types of cancers, with macrophage in 21 types of cancers and with neutrophil in 30 types of cancers, but significantly negatively correlated with CD4+ T cell in five types of cancers, with CD8+ T cell in THCA, and with dendritic cell in SARC. Furthermore, we observed that YTHDC2 was closely associated with the expression of immune checkpoint‐related genes in most types of cancers except for CESC and TGCT. In addition, YTHDC2 expressed differently in immune subtypes of cancers, including BLCA, BRCA, KIRC, LIHC, LUAD, PAAD, SKCM, STAD and UCEC. The result suggests that YTHDC2 may have an important influence on the immune infiltration in most malignant tumours. Thus, research on the value of YTHDC2 in immune infiltration from a distinct immune subtype in individual tumours may give assistance to provide new thinking of tumour immunotherapy.

It remains unknown whether YTHDC2 expression is associated with the diagnosis of cancers. Here, we set out to investigate the diagnostic value of YTHDC2 in cancers. As a result, YTHDC2 presented a high diagnostic value for seven types of cancers, including CHOL, LUSC, THCA, OV, SKCM, TGCT and UCS. It is worth pointing out that YTHDC2 may be a remarkable diagnostic biomarker for CHOL, OV, TGCT and UCS, due to its higher sensitivity and specificity. Hence, further studies need to explore the early diagnostic value of YTHDC2 in the above cancers, given essential clinical implications of YTHDC2. Concomitantly, we wonder whether YTHDC2 takes an essential part in the prognosis of cancers as well. The results demonstrated that YTHDC2 expression was closely related to the survival prognosis of LGG, READ and SKCM, involving OS, DSS and PFI. Therefore, the analysis of prognosis in cancers provides a bioinformatics basis for further experimental research on the prognostic value of YTHDC2.

Pan‐cancer analysis has emerged as an important research focus for tumorigenesis and development in recent years, our study presents the significance of YTHDC2 from the perspective of pan‐cancer, including gene expression, genetic alteration, molecular mechanism, immune infiltration, diagnostic value and clinical prognosis, suggesting YTHDC2 may be a promising biomarker for diagnosis and prognosis in certain cancers, and targeting YTHDC2 may provide new thinking to the immunotherapy of individual cancers. Although we identify the different roles of YTHDC2 in cancers from the perspective of bioinformatics, there are also several limitations in the present study. Firstly, data analysed in our study only come from the TCGA database and the GTEX database without any other database or actual clinical data. Secondly, further molecular regulatory mechanism of YTHDC2 affecting cancer initiation and progression remains unknown. Broad exploration and deep verification of molecular biology experiments are still need to perform and investigate in the individual cancer study, due to the heterogeneity and complexity of tumours. Furthermore, diverse computational methods can provide novel insights of bioinformatics exploration, such as lncRNA‐miRNA interaction predictions,^42^, ^43^, ^44^ the identification of microRNA combinatorial biomarkers^45^, ^46^, ^47^ and predictive model.^48^, ^49^ In summary, our study contributes to uncovering cancer promoting or suppressing effects of YTHDC2 in various cancer types comprehensively and provides evidence on the role of YTHDC2 in tumour cell immune infiltration, diagnostic value and clinical prognosis.

## CONFLICT OF INTEREST

The authors declare that there is no conflict of interests.

## AUTHOR CONTRIBUTIONS

**Chiyuan Zhang:** Conceptualization (lead); Data curation (lead); Investigation (lead). **Cuishan Guo:** Data curation (supporting); Formal analysis (supporting); Methodology (supporting). **Yan Li:** Data curation (supporting); Methodology (supporting). **Ling Ouyang:** Data curation (supporting); Methodology (supporting); Supervision (supporting). **Qi Zhao:** Software (equal); Writing‐original draft (equal). **Kuiran Liu:** Methodology (equal); Supervision (lead); Writing‐original draft (equal).

## CONSENT TO PARTICIPATE

Not applicable.

## CONSENT FOR PUBLICATION

Not applicable.

## Supporting information

Fig S1

Fig S2

Fig S3

Fig S4

Fig S5

Fig S6

Fig S7

Fig S8

Supplementary Material

Supplementary Material

## Data Availability

Publicly available datasets were analysed in this study, which can be found in UCSC XENA (https://xenabrowser.net/datapages/).
