# Supplementary figures and images for: The role of YTH domain containing 2 in epigenetic modification and immune infiltration of pan‐cancer
Source: J Cell Mol Med. 2021 Jul 27;25(18):8615–27. doi: 10.1111/jcmm.16818 (PMC8435423; doi:10.1111/jcmm.16818)

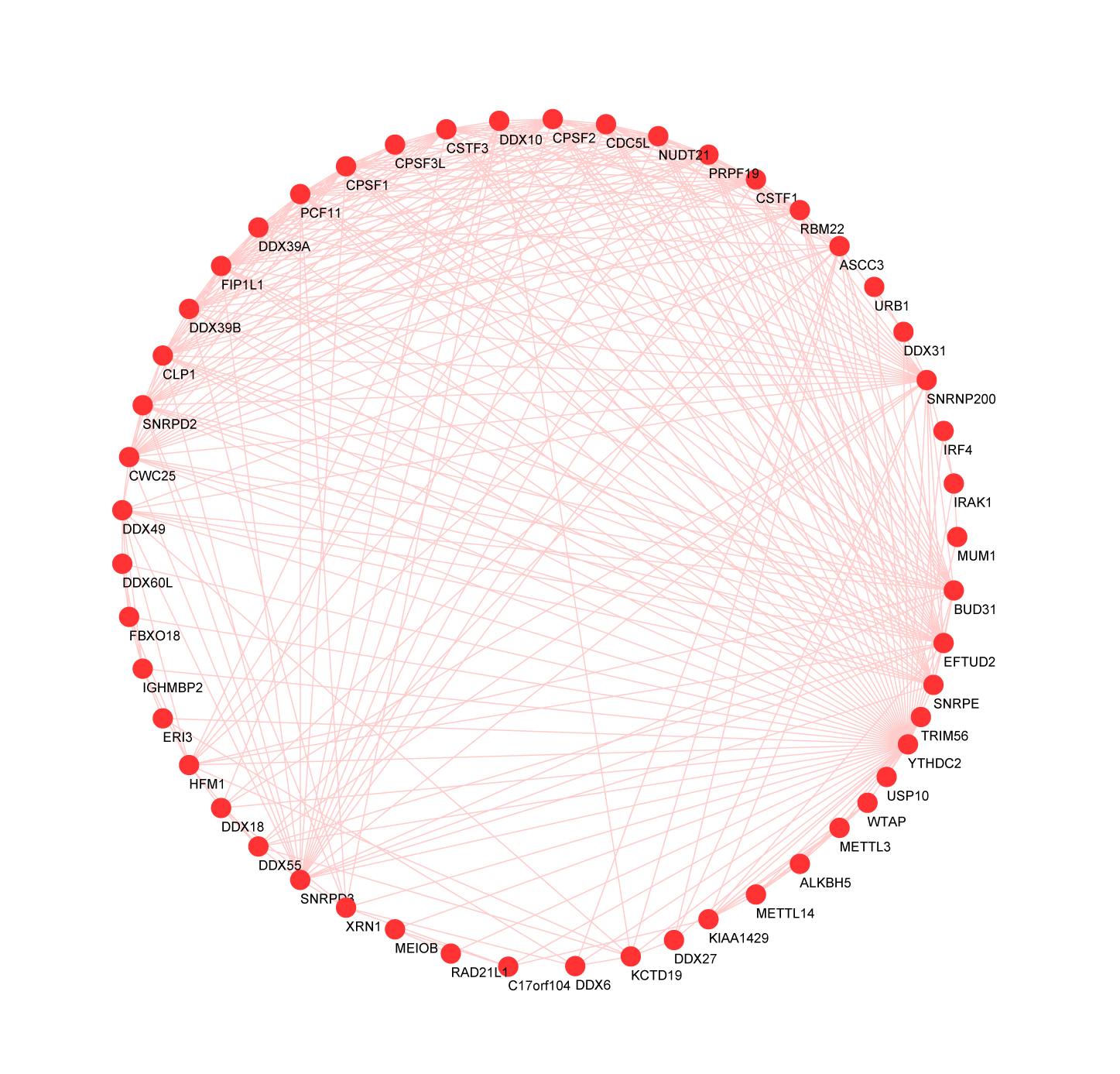

Supplement: Supplementary file 1 — Fig S1 [file JCMM-25-8615-s005.tif]

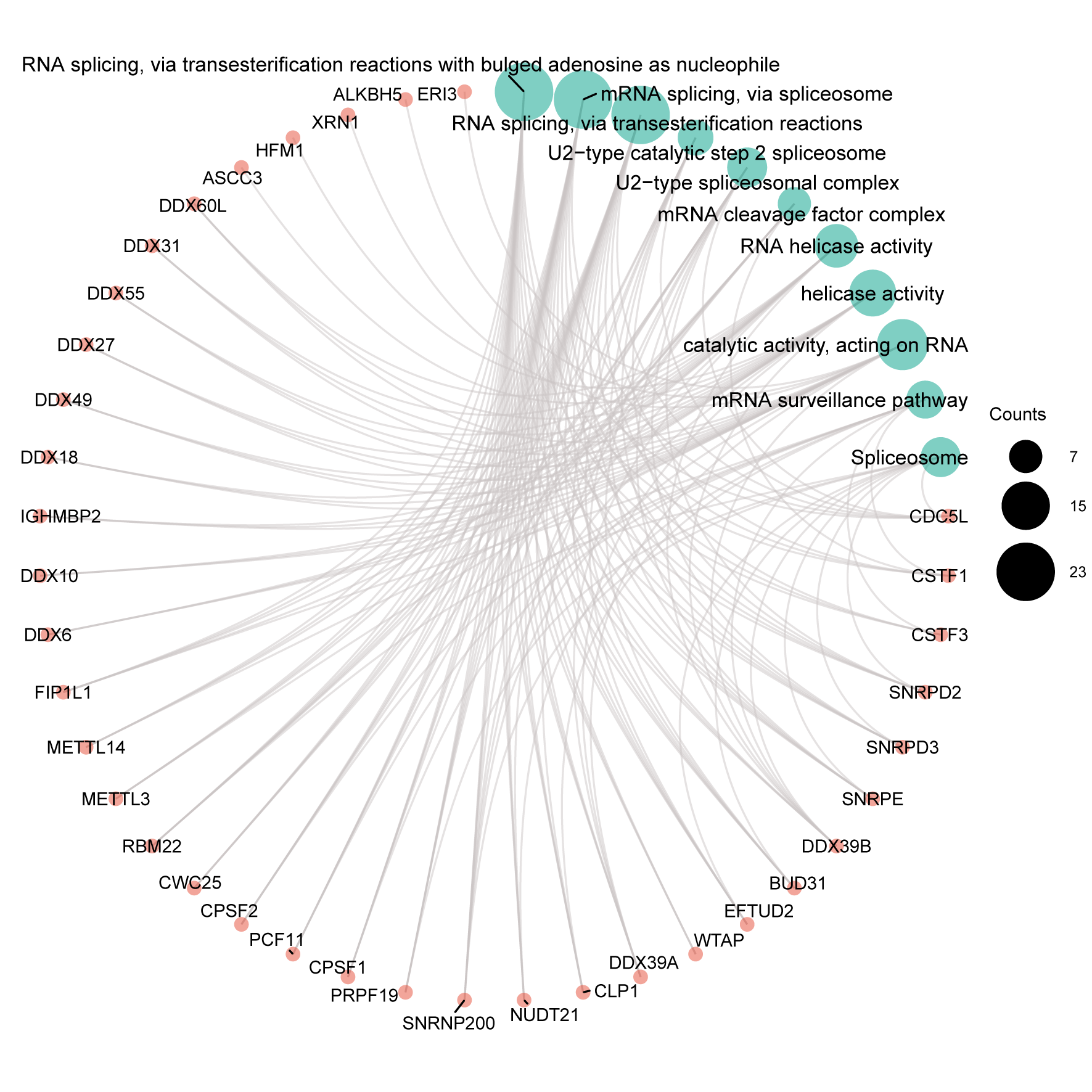

Supplement: Supplementary file 2 — Fig S2 [file JCMM-25-8615-s001.tif]

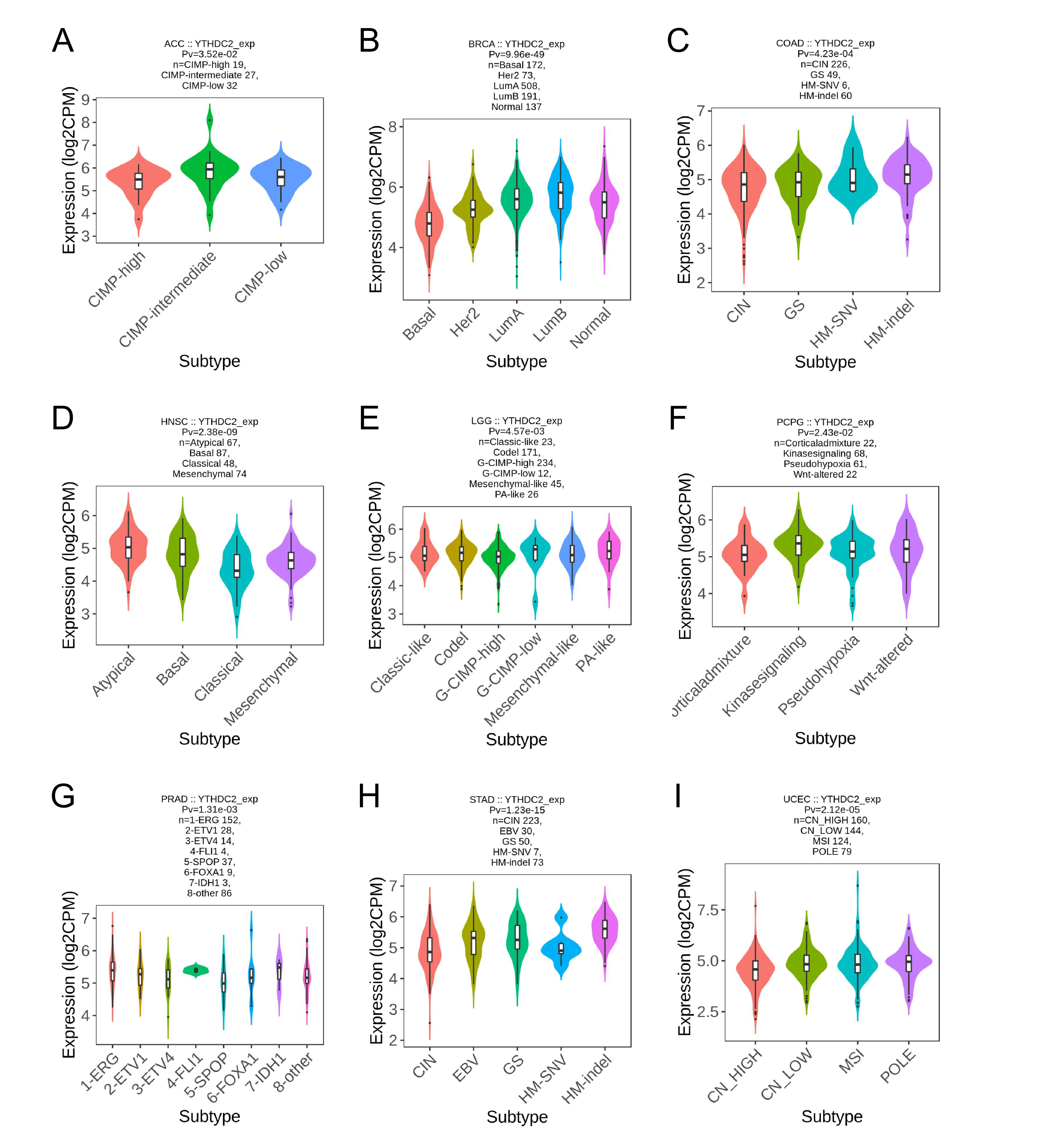

Supplement: Supplementary file 3 — Fig S3 [file JCMM-25-8615-s010.tiff]

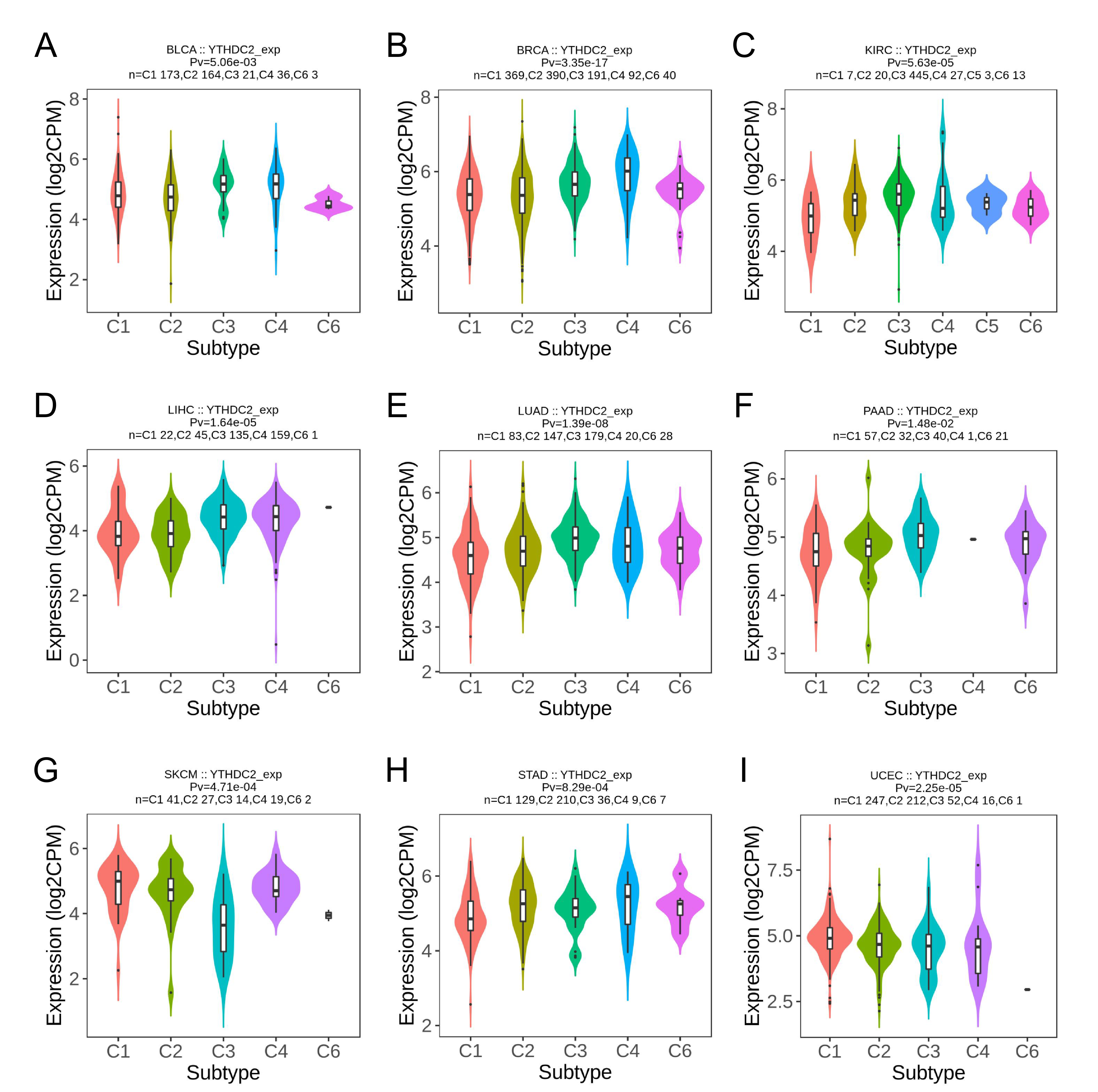

Supplement: Supplementary file 4 — Fig S4 [file JCMM-25-8615-s007.tiff]

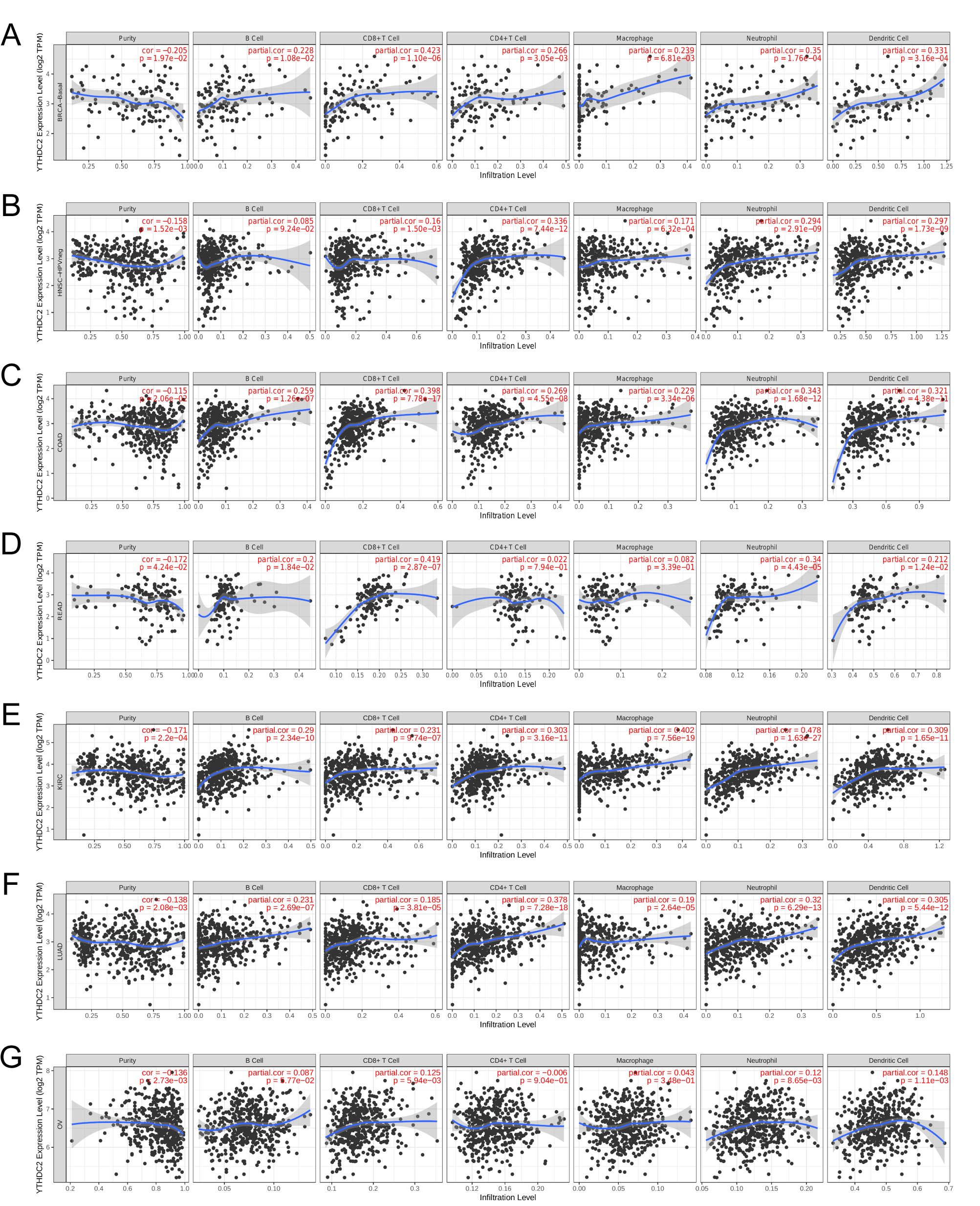

Supplement: Supplementary file 5 — Fig S5 [file JCMM-25-8615-s004.tif]

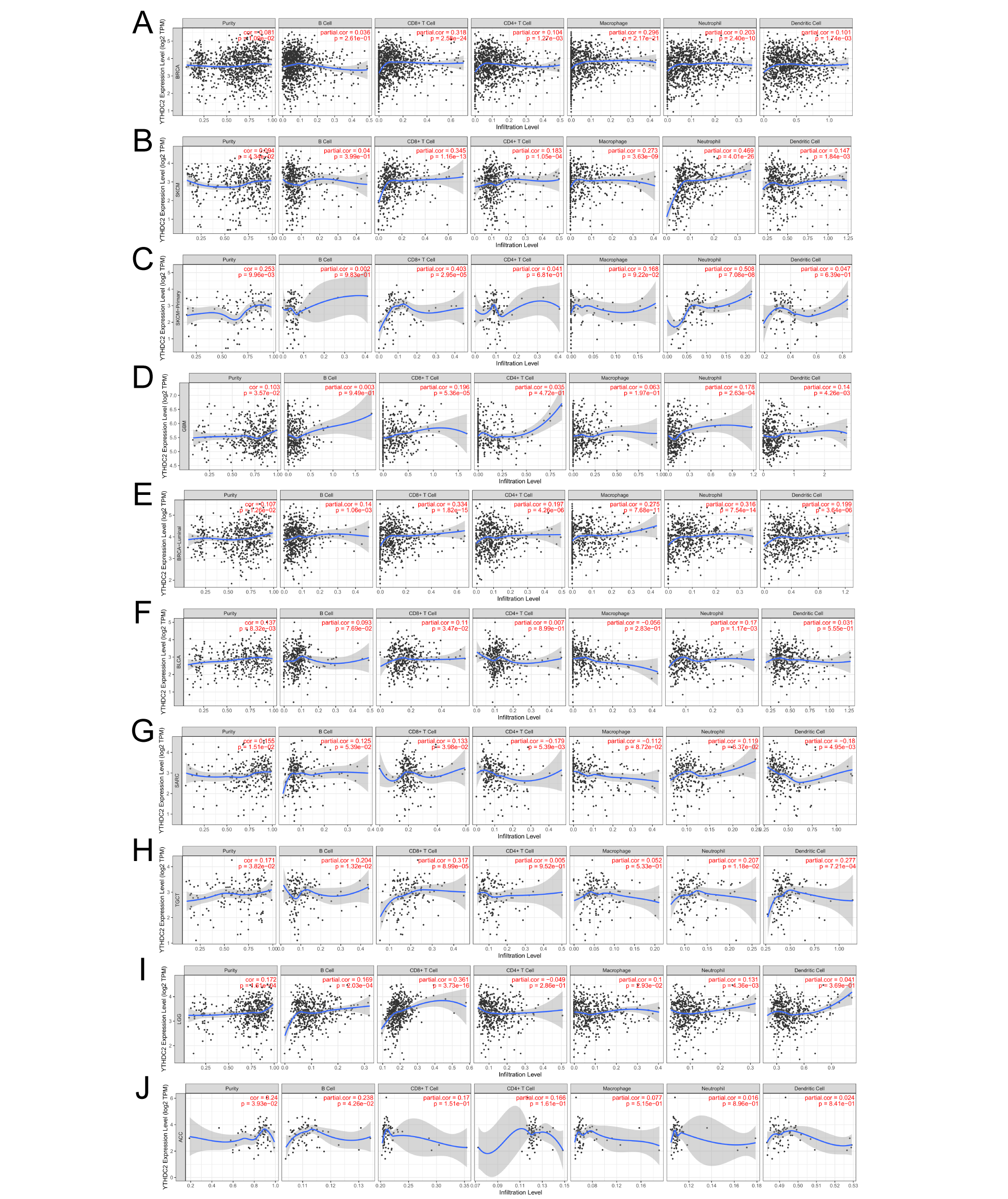

Supplement: Supplementary file 6 — Fig S6 [file JCMM-25-8615-s002.tif]

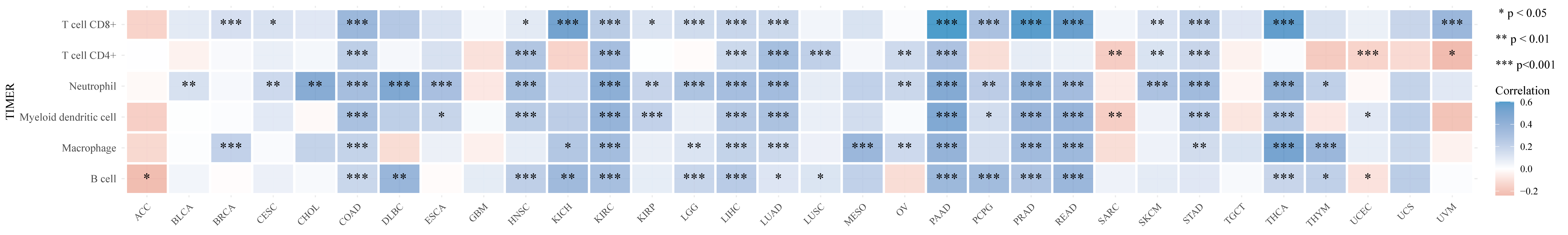

Supplement: Supplementary file 7 — Fig S7 [file JCMM-25-8615-s006.tif]

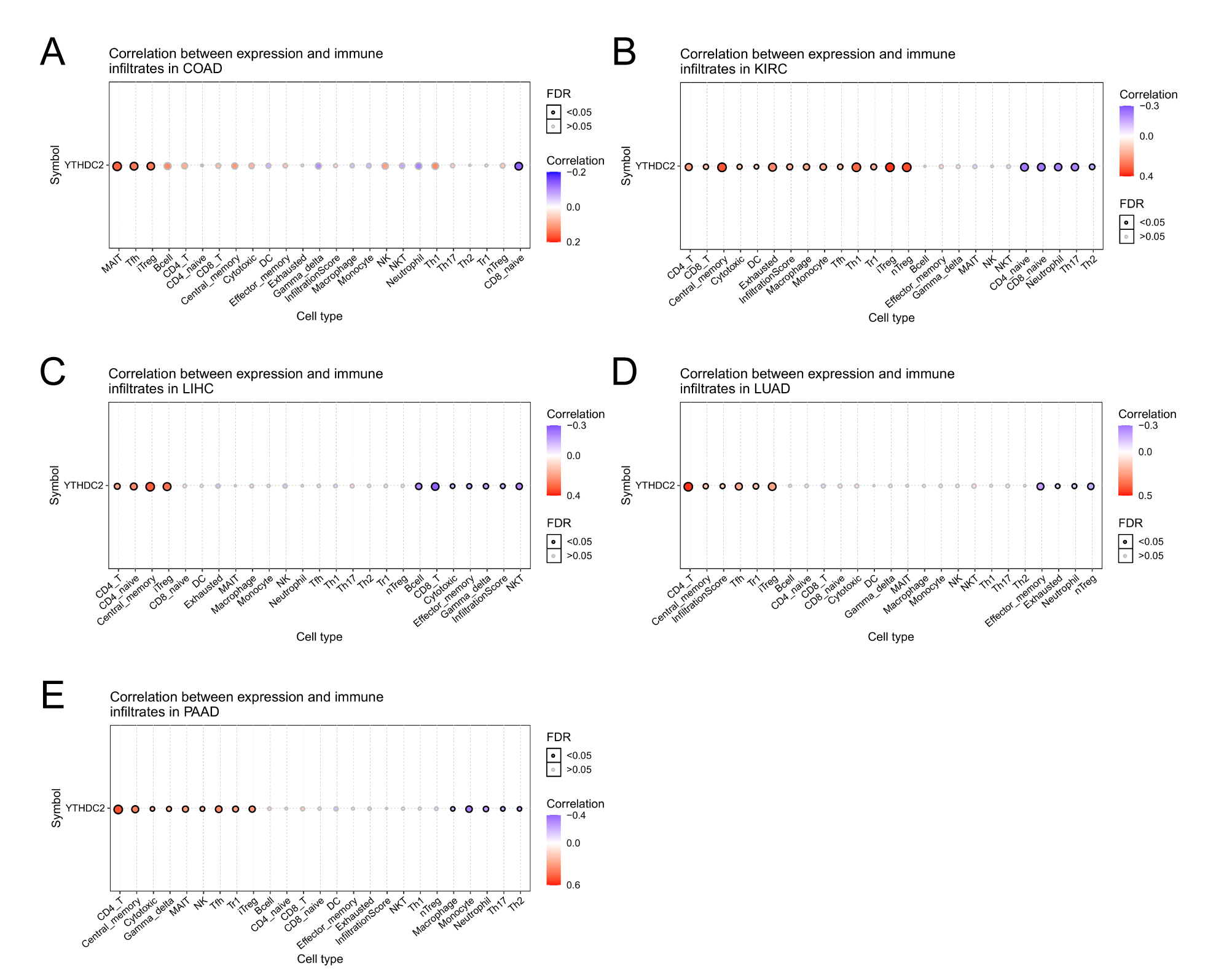

Supplement: Supplementary file 8 — Fig S8 [file JCMM-25-8615-s009.tif]
