# Supplementary material for: The role of YTH domain containing 2 in epigenetic modification and immune infiltration of pan‐cancer
Source: J Cell Mol Med. 2021 Jul 27;25(18):8615–27. doi: 10.1111/jcmm.16818 (PMC8435423; doi:10.1111/jcmm.16818)
Supplement: Supplementary file 10 — Supplementary Material [file JCMM-25-8615-s003.docx]

Figure S1. PPI network of 50 targeted binding proteins of YTHDC2.

Figure S2. Visual network of GO and KEGG analysis

Figure S3. Associations betweenYTHDC2 expression and molecular subtypes across human cancers. (A) ACC. (B) BRCA. (C) COAD. (D) HNSC. (E) LGG. (F) PCPG. (G)PRAD. (H) STAD. (I) UCEC.

Figure S4. Associations between YTHDC2 expression and immune subtypes across human cancers. (A) BLCA. (B) BRCA. (C) KIRC. (D) LIHC. (E) LUAD. (F) PAAD. (G)SKCM. (H) STAD. (I) UCEC.

Figure S5. Correlations between YTHDC2 expression and immune infiltration levels of B Cell, CD8+ T Cell, CD4+ T Cell, Macrophage, Neutrophil, and Dendritic Cell in 7 types of cancers negatively associated with tumor purity. (A) BRCA-Basal. (B) READ. (C) COAD. (D) KIRC. (E) HNSC-HPVneg. (F) LUAD. (G) OV.

Figure S6. Correlations between YTHDC2 expression and immune infiltration levels of B Cell, CD8+ T Cell, CD4+ T Cell, Macrophage, Neutrophil, and Dendritic Cell in 10 types of cancers positively associated with tumor purity. (A) BRCA. (B) SKCM. (C) SKCM-Primary. (D) GBM. (E) BRCA-Luminal. (F) BLCA. (G) SARC. (H) TGCT. (I) LGG. (J) ACC.

Figure S7. Spearman correlation analysis heat map of immune score and YTHDC2 expression. **p* < 0.05, ***p* < 0.01, ****p*< 0.001), and the asterisk represents the importance (**p*). The significance of the two groups of samples passed the Wilcox test.

Figure S8. Correlations between YTHDC2 expression and immune infiltrates of 5 types of tumors in the GSCA database. (A) COAD. (B) KIRC. (C) LIHC. (D) LUAD. (E) PAAD.
